# Hepatic tuberculosis with lower gastrointestinal symptoms mimicking hepatic metastasis: A rare case report

**DOI:** 10.1016/j.ijscr.2023.109192

**Published:** 2023-12-28

**Authors:** Farhan Shahzad, Arsalan Shah Roghani, Faizan Shah Roghani, Zaryab Ali Shah, Komal Fatima, Inam Ullah

**Affiliations:** a Khyber Teaching Hospital, Peshawar, Pakistan; b Hayatabad Medical Complex, Peshawar, Pakistan

**Keywords:** Abdominal symptoms, Diagnostic challenges, Biopsy, Necrotizing granulomatous inflammation, Hepatic tuberculosis

## Abstract

**Introduction and importance:**

The co-occurrence of *Mycobacterium tuberculosis* (MTB) affecting both the lungs and abdominal viscera is quite common, but instances of isolated Hepatic tuberculosis (TB) without concurrent clinical signs of TB are exceedingly rare.

**Case presentation:**

We present a case of a 55-year-old woman who complained of abdominal pain, weight loss, fever and changes in bowel habits. A definitive diagnosis of hepatic TB was made through microscopic examination, revealing necrotizing granulomatous inflammation accompanied by caseous necrosis. The patient received antitubercular therapy without experiencing any noticeable side effects during follow-up.

**Clinical discussion:**

Hepatic TB without active pulmonary TB is quite rare case. The patient presents with abdominal pain, fever, weight loss and jaundice. The diagnostic process includes CT (computerize tomography) imaging and subsequent biopsy to confirm it histopathologically. Following the same approach, we did biopsy from the targeted hepatic lesion that showed hepatic tuberculosis. In most cases it is treated with anti-tubercular drugs. However, some complicated cases might need surgical intervention.

**Conclusion:**

This case report highlights the significance of considering TB into account as a potential cause in patients with lower gastrointestinal symptoms in TB endemic areas by emphasizing the diagnostic challenges posed by hepatic tuberculosis with isolated liver involvement. In order to prevent serious complications of abdominal TB, early diagnosis and timely treatment is crucial.

## Highlights

• Hepatic tuberculosis without pulmonary involvement is rare.
• Patient presents with right hypochondrial pain, weight loss, fever and altered bowel habits.
• Ultrasound and CT scan help in diagnosis which. is confirmed through biopsy from hepatic lesion
• Definitive treatment is quadruple therapy with anti-tubercular drugs consisting of isoniazid (INH), rifampicin (RMP), pyrazinamide (PZA), and ethambutol (EMB).
• Isolated hepatic tuberculoma or liver abscess might need surgical intervention.

## 1 Introduction and importance

The study adheres to the SCARE criteria in its reporting [1]. Mycobacterium poses a widespread global health challenge, notably affecting regions like the Indian subcontinent [2]. While developed countries have seen an uptick in cases due to secondary immunosuppression caused by conditions such as cancer and HIV (human immunodeficiency virus) infections, developing nations grapple with tuberculosis primarily due to poor socioeconomic conditions [3]. This disease's significant impact on developing nations results in a staggering death toll of approximately 2,000,000 each year [4]. While the lungs are the primary focus, other body parts can also be affected, with the abdomen being particularly susceptible (at rates ranging from 6 % to 38 %) [2]. Abdominal visceral tuberculosis is a usual health issue in the Indian subcontinent, affecting multiple organs including liver [3].

Hepatic TB is often associated with pulmonary involvement, though cases of isolated hepatic TB without active pulmonary or miliary TB are exceedingly rare and primarily documented in case reports and series within English literature [3]. Incidence estimates for hepatic TB stand at around 1 % of all active TB cases, and isolated liver TB accounts for <1 % of total cases [5]. The infrequency of hepatic TB is attributed to the liver's low oxygen tension, rendering the environment unfavorable for the growth of *Mycobacterium tuberculosis* [2,4]. The liver's engagement in both pulmonary and extrapulmonary tuberculosis often remains without noticeable clinical manifestations. In this context, we present a case of 55-year-old woman with lower gastrointestinal symptoms, where hepatic TB is detected. This case emphasizes the importance of considering, evaluating, and excluding tuberculosis as a potential cause in patients presenting with liver lesions. Accurately diagnosing liver TB holds great significance, as untreated abdominal TB carries a 50 % mortality rate [6]. Furthermore, it's crucial to note that this is a treatable ailment, and swift diagnosis along with timely intervention can prevent severe consequences.

## 2 Case presentation

We are presenting a case involving a 55-year-old woman who had no known pre-existing health conditions or any family related disorders or significant contact history with tuberculous patients. She was admitted to our hospital through emergency department due to pain in the right upper abdomen, altered bowel habits, weight loss, and fever for one year. The abdominal pain developed gradually, occurred at irregular intervals, and was characterized by a dull sensation that did not radiate to other areas. Her fever started slowly, had a mild intensity, was constant, and came with episodes of shivering and chills. Upon conducting a physical examination, it was observed that the patient's abdomen was soft and not visibly swollen. There was mild tenderness experienced in the right upper abdominal region. The patient had a pulse rate of 84 beats per minute, a temperature of 99 °F and a blood pressure of 125/85 mmHg.

Upon analyzing the laboratory results, hemoglobin level was 9.7 g/dl, the hematocrit measured 29.8 %, the count of white blood cells was 18.83 × 10^3^/μl. Additionally, her serum CRP (C-reactive protein) level was determined to be 131.41 mg/dl, alkaline phosphatase and lactate dehydrogenase had values of 337 U/l and 441 U/l respectively. An assessment of stool for occult blood revealed a positive result, while the rest of the baseline laboratory tests fell within the normal range as indicated in Table 1.

**Table 1:** Laboratory parameters and radiological imaging reports of the patient.

S. no	Investigation	Finding	Normal range	Remarks
1	WBCs	18.83 × 10^3^/μl	4–11	High
2	Neutrophils	78.3 %	40–75 %	High
3	Lymphocytes	14 %	20–45 %	Low
4	Hemoglobin	9.7 g/dL	11.5–17.5	Low
5	HbsAg/Anti-HCV/Anti-HIV	Negative		
6	RBS	100.6 mg/dL	70–140	Normal
7	Albumin	3.6 g/dL	3.5–5.0	Normal
8	Total bilirubin	0.3 mg/dL	0.1–1.0	Normal
9	ALT/GPT	16 units/L	10–50	Normal
10	ALP	337 units/L	35–104	High
11	LDH	441 units/L	91–180	High
12	Serum CRP	131.41 mg/L	<5.0	High
13	Blood ESR	63 mm/1st hr.	0–30	High
14	AFP	1.46 IU/ml	<5.0	Normal
15	Stool for occult blood	Positive		
16	CT abdomen/pelvis with oral and IV contrast	Multiple hypodense lesions in liver		Largest 2.8 cm
		Paraaortic lymphadenopathy		Largest 2.1 cm
17	Colonoscopy	No growth/polyps/strictures or ulcerations seen		
18	Trucut biopsy of right hepatic region and Laparoscopic biopsy of hepatic lesion	Necrotizing granulomatous inflammation		Caseous necrosis

**Fig. 1:**
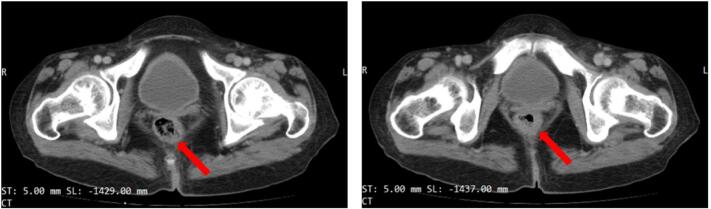
CT scan showing thickened rectal wall.

**Fig. 2:**
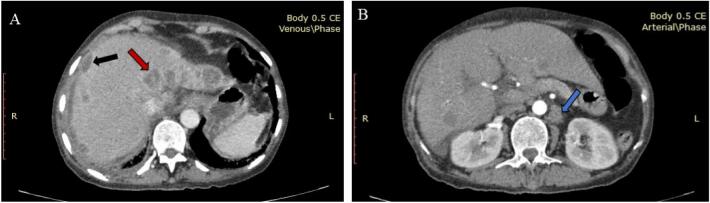
CT scan showing subcapsular deposits-frosted liver (black arrow) and parenchymal deposits (red arrow) (A). Para-aortic lymphadenopathy (blue arrow) (B). (For interpretation of the references to colour in this figure legend, the reader is referred to the web version of this article.)

The abdominopelvic ultrasound revealed multiple solid iso-hypoechoic lesions in right and left lobes of the liver, varied in size ranging from a few millimeters to 30 mm (3 cm). An abdominal and pelvic CT scan showed extensive non-enhancing hypodense nodules within both lobes of the liver, ranged in size from 1 to 3 cm, and thickened rectal wall (Fig. 1). Another triphasic CT scan revealed subcapsular deposits, parenchymal deposits and para-aortic lymphadenopathy (Fig. 2).

Due to the concerning findings and presentation of the patient, there was a suspicion of rectal carcinoma. To investigate this possibility further, various tumor markers were assessed, including alpha-fetoprotein (AFP), carcinoembryonic antigen (CEA), and carbohydrate antigen 19-9 (CA 19-9). All of these tumor marker assessments, however, revealed negative outcomes.

**Fig. 3:**
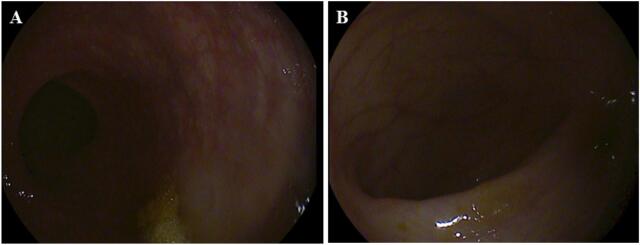
Colonoscopy images showing normal rectum.

**Fig. 4:**
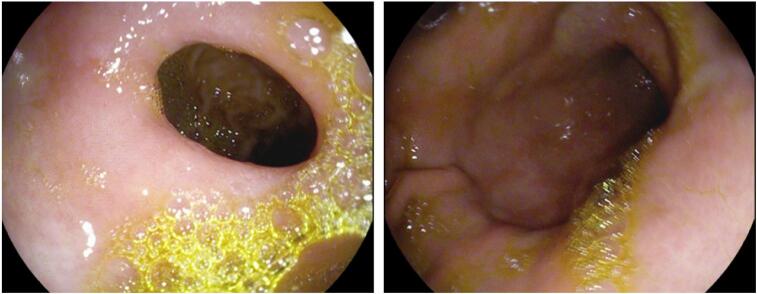
Endoscopy (normal).

A colonoscopy was performed out in order to check for rectal carcinoma, if it developed. The entire course of the large intestine, up to the cecum, was thoroughly investigated during the colonoscopy, however, no obvious signs of strictures, polyps, aberrant growths, or ulcerations were seen (Fig. 3). Therefore, rectal cancer as a probable cause for the patient's condition was screened out. We conducted an upper endoscopy to investigate the cause of anemia in the upper gastrointestinal system. However, the procedure did not reveal any pathology (Fig. 4).

**Fig. 5:**
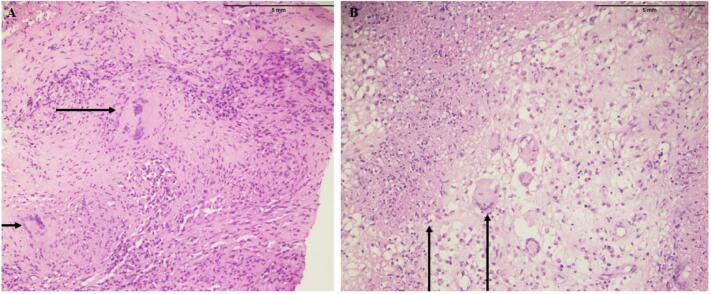
The examined sections of the liver tissue display distorted architecture, showing numerous granulomas. These granulomas consist of epithelioid cells, which are surrounded by infiltrating lymphocytes and Langhan's multinucleated giant cells. Moreover, areas of tissue necrosis and fibrosis are seen.

An ultrasound-guided liver biopsy from the right hepatic area was taken whose results were surprising since they showed a pattern of necrotizing granulomatous inflammation with caseous necrosis (Fig. 5A). However, the ZN (Ziel-Neelsen) staining did not show presence acid-fast bacilli. Additionally, a Gomori Methenamine Silver (GMS) staining also revealed the absence fungi. The unexpected result of liver biopsy shifted attention to the potential of hepatic TB as the underlying cause of the patient's disease.

A multidisciplinary team (MDT), consisting up of a range of healthcare professionals comprising general physicians, general surgeons, diagnostic and interventional radiologists, as well as experts in hepatobiliary surgery and laparoscopic procedures, after thoughtful deliberation, decided to proceed forward with a diagnostic laparoscopy. During laparoscopy, two tiny biopsies were taken from the liver nodules whose confirmed the results of the initial biopsy, which had previously demonstrated the existence of a necrotizing granulomatous inflammation with an accompanying caseous necrosis (Fig. 5B).

The diagnosis of diffuse hepatic TB was confidently made on the basis of the similar findings across these samples. The histological examination, which continues to be the most accurate method for diagnosing abdominal visceral TB, was used as the foundation for this result due to its strong evidence [2].

Effective management of hepatic TB involves a quadruple therapy regimen consisting of isoniazid (INH), rifampicin (RMP), pyrazinamide (PZA), and ethambutol (EMB), which mirrors the treatment protocol for other forms of extra pulmonary TB [7]. Administering this quadruple therapy for a duration of one year is recommended due to the increasing incidence of drug-resistant TB strains. Notably, clinical improvement, including the resolution of hepatomegaly, restoration of appetite, reduction in fever, and regaining lost weight, is typically observed within a span of 2 to 3 months after treatment initiation [8]. As the weight of our patient was 55 kg, so she was started on 4 drugs regimen for 2 months (IP), 4 tablets per day. Then, 3 drugs regimen for 4 months (CP) according to reported literature (Table 2) [9]. She was followed up for 2 times after discharge, each 2 months apart, with improvement in her symptoms and no adverse effects of antitubercular drugs were observed.

**Table 2:** Treatment details of tuberculosis.

S. no	Weight category (total body weight in kg)	Intensive phase (IP) (2 months) INH/RMP/PZA/EMB 75/150/400/275 (in mg)	Continuation phase (CP) (4 months) INH/RMP/EMB 75/150/275 (in mg)
1	25–39	2 tablets	2 tablets
2	40–54	3 tablets	3 tablets
3	55–69	4 tablets	4 tablets
4	≥70	5 tablets	5 tablets

Surgery for hepatic tuberculosis is reserved for cases where isolated hepatic tuberculoma or liver abscess doesn't respond to anti-tuberculosis medications. Procedures like enucleation, local excision, abscess drainage, or biliary drainage are used in less complicated cases, while liver segmentectomy and hemi hepatectomy are considered for more complex situations. Timely diagnosis and treatment of TB are crucial to prevent the need for major hemi hepatectomy, associated with high morbidity and mortality [10].

The limitation of this case report is we did not capture intraoperative findings during laparoscopy.

## 3 Clinical discussion

*Mycobacterium tuberculosis* (MTB) primarily affects the lungs but can also spread to various organs, most commonly to the abdomen [5] where it often targets different viscera, including the liver. While MTB constitutes 2 % of active tuberculosis (TB) cases in immunocompetent individuals, this proportion can rise significantly to 16 %–78 % in cases where patients have associated hematological malignancies. Factors contributing to the risk of MTB include immunosuppressive conditions like AIDS (acquired immunodeficiency syndrome), miliary presentations (which make up around 50 % of cases), diabetes mellitus, use of immunomodulatory or cytotoxic drugs, organ transplants, and specific cancers like head and neck cancers, along with other hematological malignancies [11]. Interestingly, our case did not exhibit any of these common risk factors.

It's notable that approximately 80 % of reported hepatic TB cases result from the systemic dissemination of acid-fast bacilli (AFB). However, cases with isolated liver involvement are exceptionally rare, accounting for <1 % of TB patients, with fewer than 100 such cases documented in the available literature [11]. The occurrence of hepatic TB without concurrent active pulmonary or miliary TB is even rarer, representing <1 % of all cases [7]. Hepatic tuberculosis (TB) manifests in three distinct forms. The most prevalent variant, affecting 50 %–80 % of patients, is Diffuse Hepatic TB, which often presents in conjunction with lung involvement or miliary TB. The second form is a rare occurrence where Diffuse Hepatic TB is observed without a primary lung focus, which aligns with the presentation in our case. The third manifestation, known as Isolated Localized Tuberculosis (ILT), represents an even rarer subtype of hepatic TB [4]. Localized hepatic tuberculosis is an infrequent ailment that primarily affects immunocompromised individuals [12].

The clinical presentation of hepatic tuberculosis (TB) tends to lack specificity. Among the most prevalent complaints are loss of appetite (64 %), weight loss (64 %), fever (50 %), and jaundice (42.3 %) [7]. However, it's important to highlight that our case did not exhibit jaundice or anorexia.

During physical exam, hepatomegaly emerges as the most common physical finding, observed in approximately 96 % of cases [2] and splenomegaly occurring in a range of 18 % to 55 % of cases [7]. It's worth noting that neither splenomegaly nor hepatomegaly was evident in our specific case. In instances of hepatic TB, there is usually an elevation in liver enzymes, including transaminases, gamma-glutamyl transferase (GGT), and alkaline phosphatase (ALP) [5]. However, in our case, elevated ALP was the only notable marker, while transaminase levels remained within the normal range.

Radiological investigations for hepatic tuberculosis (TB) often mirror those conducted for more common diseases, making diagnosis challenging. Ultrasonography frequently reveals hepatic masses that are either hypoechoic or, less commonly, hyperechoic or solid in nature [7]. In our case, multiple iso-hypoechoic solid lesions were identified in both liver lobes. Specific radiological indicators for hepatic TB are lacking. Nevertheless, certain signs such as the “target sign” (characterized by rim-enhancement of tuberculomas with caseous necrosis) and the “cluster sign” (involving the merging of small tuberculomas to form an abscess) could suggest the presence of hepatic TB. Regrettably, these particular findings were not evident in our case. In the context of hepatic TB, CT scans tend to display hypodense lesions, which aligns with our case. However, it's important to consider that these hypodense lesions can be indicative of a range of other conditions as well, including fungal diseases, sarcoidosis, lymphomas, leukemia, and other granulomatous diseases like brucellosis [5].

Relying solely on imaging findings, it's challenging to distinguish these diffuse hepatic lesions resembling metastases from other masses such as hepatocellular carcinoma (HCC), Hodgkin lymphomas, or metastatic growths. Often, these lesions are misidentified as primary or metastatic carcinoma [6]. In our case, the condition was mistakenly diagnosed as rectal carcinoma with hepatic metastasis. Imaging studies have categorized hepatic TB into three main types: tuberculous cholangitis, parenchymal tuberculosis, and serohepatic tuberculosis [13]. In instances of serohepatic TB, a distinct pattern becomes evident. Multiple hypodense lesions are found in the outer regions of the liver, particularly in the subscapular area. These lesions are encircled by a thickened and enhanced liver capsule and sub capsule, resembling a “sugar coating.” This unique presentation is commonly referred to as a “frosted liver” (Fig. 2A). It's important to emphasize that serohepatic tuberculosis is the least prevalent form among the various types of hepatic tuberculosis [5,13].

Even with the utilization of a triphasic CT scan and contrast-enhanced ultrasound, correctly diagnosing hepatic TB without pulmonary involvement remains problematic. It's advisable to conduct a comprehensive CT scan covering the abdomen, pelvis, and thorax to effectively rule out potential TB sites elsewhere in the body. Moreover, neither CT scans nor ultrasounds inherently raise suspicion regarding TB [11]. While hepatic TB cases with active pulmonary or miliary TB can be diagnosed using percutaneous fine needle biopsy, the scenario becomes more complex when hepatic TB occurs without concurrent active pulmonary or miliary TB. In such instances, ultrasound-guided or CT-guided biopsies, or laparoscopic biopsies, are necessary [8]. In our specific case, we followed this approach and performed both ultrasound-guided and laparoscopic biopsies, both yielding consistent results-necrotizing granulomatous inflammation with caseous necrosis.

The unique and nonspecific presentation of hepatic TB makes diagnosis challenging through non-invasive methods, including imaging. This is further complicated by the fact that other localized liver deposits, such as metastases, can present with similar imaging characteristics. Consequently, histopathological examination of a biopsy specimen from the liver lesion remains the gold standard for hepatic TB diagnosis [2]. Notably, the presence of caseating granulomas is a prominent and diagnostic feature for identifying MTB during biopsy [2,5,8].

Histopathological assessments of hepatic TB typically reveal epithelioid granulomas in 80 %–100 % of cases, caseating necrosis in 30 %–83 % of cases, and the presence of acid-fast bacilli (AFB) on smear examination in 0 %–59 % of cases [7]. In our case, hepatic deposits displayed epithelioid granulomas and caseous necrosis, while AFB were not detected on smear examination.

The accuracy of the diagnosis is of utmost importance, given the significant consequences of untreated abdominal TB [6]. To expedite the diagnostic process, it's essential to consider the possibility of disseminated TB, as doing so might lead directly to the correct diagnosis. This approach can substantially reduce both the patient's morbidity and mortality [11]. Once a definitive diagnosis is established, the primary goal is to initiate treatment as promptly as possible, as this intervention can lead to lesion resolution within a span of 6 to 12 months [4].

## 4 Conclusion

To conclude, when presented with nonspecific lower gastrointestinal symptoms in a patient without apparent pulmonary TB, the consideration of a TB infection becomes crucial. This is particularly relevant in developing countries where TB prevalence is notably high. It's prudent to also factor in the possibility of hepatic TB when contemplating differential diagnoses. Incorporating hepatic TB into the diagnostic process serves to minimize unnecessary medical interventions for patients and eases the strain on investigative resources, a significant advantage in resource constrained settings. Timely diagnosis and treatment of TB are vital to avoid surgery, linked with elevated morbidity and mortality risks.

## Consent

Written informed consent was obtained from the patient for publication and any accompanying images. A copy of the written consent is available for review by the Editor-in-Chief of this journal on request.

## Ethical approval

Study was approved by Institutional Research and Ethical Review Board of Khyber Medical College Peshawar accordance with declaration of Helsinki (2013).

IREB No: 604.

Date: 25th September 2023.

## Funding

No funding source or sponsor.

## Author contribution

1. Writing manuscript, Final Drafting, Proof Reading: Farhan Shahzad (Main author)
2. Writing the paper: Arsalan Shah Roghani (Co-first Author)
3. Data collection: Faizan Shah Roghani (Co Author)
4. Data collection and proof reading: Zaryab Ali Shah (Co Author)
5. Data collection and proof reading: Komal Fatima (Co Author)
6. Proof reading: Inam Ullah (Co Author)

## Guarantor

Farhan Shahzad.

## Research registration number

N/A.

## Conflict of interest statement

No conflict of interest.
